# Supplementary material for: Serovar-level Identification of Bacterial Foodborne Pathogens From Full-length 16S rRNA Gene Sequencing
Source: bioRxiv. 2023 Jun 28:2023.06.28.546915. Preprint. [Version 1] doi: 10.1101/2023.06.28.546915 (PMC10327058; doi:10.1101/2023.06.28.546915)
Supplement: Supplement 1 [file NIHPP2023.06.28.546915v1-supplement-1.pdf]

bioRxiv preprint doi: https://doi.org/10.1101/2023.06.28.546915; this version posted June 28, 2023. The copyright holder for this preprint (which was not certified by peer review) is the author/funder, who has granted bioRxiv a license to display the preprint in perpetuity. It is made available under aCC-BY 4.0 International license.

Supplemental Tables and Figures

Serovar	Total in Group	Correct (%)	Indeterminate (%)	Incorrect (%)
Enteritidis	300	99.3	0	0.7
Typhimurium/Monophasic	288	95.1	2.4	2.4
Typhi	101	89.1	10.9	0
Infantis	60	98.3	1.7	0
Newport	54	88.9	1.9	9.3
Heidelberg	41	100	0	0
Kentucky	41	97.6	2.4	0
Anatum	36	94.4	5.6	0
Bareilly	34	85.3	8.8	5.9
Agona	33	97	3	0
Saintpaul	32	90.6	3.1	6.3
Indiana	29	79.3	10.3	10.3
Montevideo	29	96.6	3.4	0
Senftenberg	27	85.2	7.4	7.4
Hadar	26	100	0	0
Javiana	21	9.5	81	9.5
Muenchen	19	94.7	0	5.3
Dublin	18	83.3	0	16.7
Reading	18	72.2	22.2	5.6
Derby	14	57.1	21.4	21.4
Goldcoast	14	92.9	0	7.1
Rubislaw	14	71.4	28.6	0
Weltevreden	14	100	0	0
Schwarzengrund	12	100	0	0
Thompson	12	66.7	25	8.3
Albany	11	100	0	0
Choleraesuis	11	81.8	9.1	9.1
Bovismorbificans	10	80	0	20
Inverness	10	100	0	0
Tennessee	10	90	10	0
Gallinarum	9	55.6	0	44.4
Oranienburg	9	77.8	0	22.2
Gaminara	8	50	25	25
London	8	87.5	12.5	0
Braenderup	7	100	0	0
I 1,4,[5],12:b:-	7	14.3	28.6	57.1
Paratyphi A	7	100	0	0
Give	6	33.3	16.7	50
Mississippi	6	100	0	0
Brandenburg	5	40	20	40
Minnesota	5	40	20	40
Muenster	5	100	0	0
Uganda	5	100	0	0
V 66:z41:-	5	100	0	0
Worthington	5	100	0	0
Bredeney	4	0	50	50
II 1,4,12,[27]:b:[e,n,x]	4	50	50	0
IIIb 60:r:z	4	100	0	0
Mbandaka	4	0	0	100
Ohio	4	100	0	0
Panama	4	50	0	50
Paratyphi B	4	75	0	25

Supplemental Table 1. Serovar assignment outcomes for all *Salmonella* serovars containing at least 4 assemblies within our reference database. Correct, incorrect, and indeterminate rates are reported for each serovar. The test queries used to generate this data included all assemblies belonging to any serovar with at least 4 entries in our reference database.

bioRxiv preprint doi: https://doi.org/10.1101/2023.06.28.546915; this version posted June 28, 2023. The copyright holder for this preprint (which was not certified by peer review) is the author/funder, who has granted bioRxiv a license to display the preprint in perpetuity. It is made available under aCC-BY 4.0 International license.

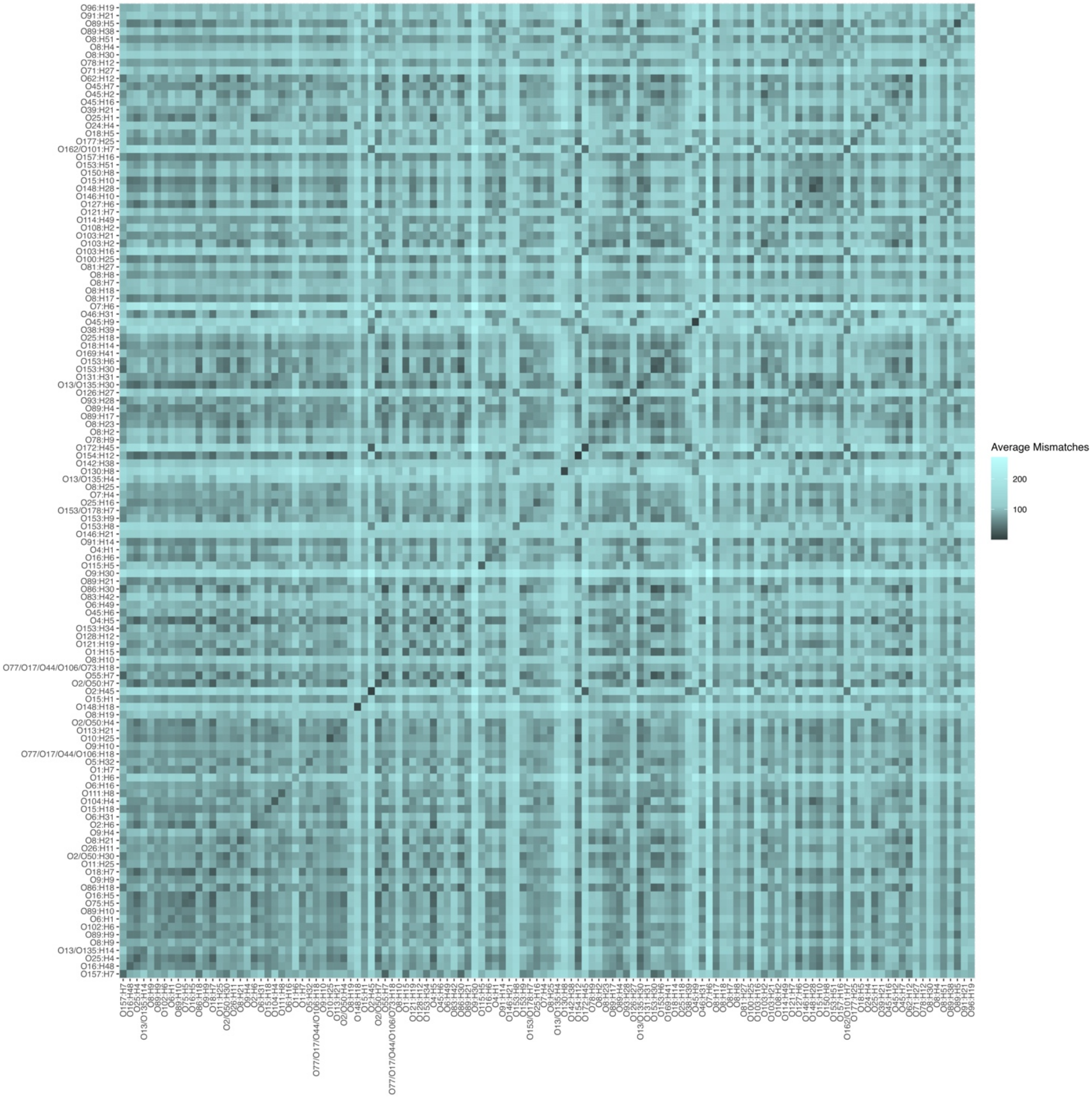

Supplemental Figure 1. Average dissimilarity between the 16S rRNA gene profiles of *E. coli* serovars. Pairwise dissimilarities were defined as the number of nucleotide mismatches between alignments of the full set of 16S rRNA genes (the 16S rRNA gene profile) of *E. coli* assemblies, after optimally re-arranging the profiles relative to one another (Methods).
